# Considerations and guidance in designing equity-relevant clinical trials

**DOI:** 10.1186/s12939-017-0591-1

**Published:** 2017-06-05

**Authors:** Lawrence Mbuagbaw, Theresa Aves, Beverley Shea, Janet Jull, Vivian Welch, Monica Taljaard, Manosila Yoganathan, Regina Greer-Smith, George Wells, Peter Tugwell

**Affiliations:** 1 Department of Health Research Methods, Evidence and Impact, McMaster University, Hamilton, ON Canada; 2 Biostatistics Unit, Father Sean O’Sullivan’s Research Centre, St Joseph’s Healthcare Hamilton, 50 Charlton Avenue East, 3rd Floor Martha Wing, Room H321, Hamilton, ON L8N 4A6 Canada; 3 Ottawa Hospital Research Institute, Center for Practice Changing Research and School of Epidemiology, Public Health and Preventive Medicine, Faculty of Medicine, University of Ottawa, Ottawa, ON K1H 8M5 Canada; 4 University of Ottawa and Ottawa Hospital Research Institute, 501 Smyth Road, Ottawa, ON K1H 8M2 Canada; 5 Bruyère Research Institute, Ottawa, Canada; 6 School of Epidemiology, Public Health and Preventive Medicine, University of Ottawa, Ottawa, Canada; 7 Ottawa Hospital Research Institute, Ottawa, Canada; 8 Clinical Epidemiology Program, Ottawa Hospital Research Institute, Ottawa, ON Canada; 9 Department of Epidemiology and Community Medicine, University of Ottawa, Ottawa, ON Canada; 10 Bruyère Research Institute, Bruyère Continuing Care and University of Ottawa, 85 Primrose, Ottawa, ON Canada; 11 Healthcare Research Associates, 2700 Concord Place, Hazel Crest, IL USA; 12 Cardiovascular Research Methods Centre, University of Ottawa Heart Institute, Ottawa, ON K1Y4W7 Canada; 13 Department of Medicine Faculty of Medicine, University of Ottawa, Ottawa, Canada; 14 WHO Collaborating Centre for Knowledge Translation and Health Technology Assessment in Health Equity, Bruyère Research Institute, Ottawa, Canada

**Keywords:** Health equity, PROGRESS plus, Randomized trial, Design, Gender, Ethnicity, Religion, Education, Socio-economic

## Abstract

Health research has documented disparities in health and health outcomes within and between populations. When these disparities are unfair and avoidable they may be referred to as health inequities. Few trials attend to factors related to health inequities, and there is limited understanding about how to build consideration of health inequities into trials. Due consideration of health inequities is important to inform the design, conduct and reporting of trials so that research can build evidence to more effectively address health inequities and importantly, ensure that inequities are not aggravated. In this paper, we discuss approaches to integrating health equity-considerations in randomized trials by using the PROGRESS Plus framework (**P**lace of residence, **R**ace/ethnicity/culture/language, **O**ccupation, **G**ender, **R**eligion, **E**ducation, **S**ocio-economic status, **S**ocial capital and “Plus” that includes other context specific factors) and cover: (i) formulation of research questions, (ii) two specific scenarios relevant to trials about health equity and (iii) describe how the PROGRESS Plus characteristics may influence trial design, conduct and analyses. This guidance is intended to support trialists designing equity-relevant trials and lead to better design, conduct, analyses and reporting, by addressing two main issues: how to avoid aggravating inequity among research participants and how to produce information that is useful to decision-makers who are concerned with health inequities.

## Background

Equity in health refers to the absence of unnecessary and avoidable differences in health that are considered to be unfair and unjust [1, 2]. Populations that experience disadvantages in opportunities for health experience health inequities, and this is reflected in poor health outcomes. There is a paucity of high quality evidence on how to reduce health inequities, and this creates challenges for decision-makers who have to consider the effects of interventions on health equity in the general population and among groups of people experiencing health inequities [3]. This paucity may be due to a lack of explicit criteria to identify vulnerable or disadvantaged populations when planning study interventions, and once such subgroups have been identified, a failure to properly accommodate them in the design, conduct and analysis of the trial [4]. The PROGRESS Plus acronym (**P**lace of residence, **R**ace/ethnicity/culture/language, **O**ccupation, **G**ender, **R**eligion, **E**ducation, **S**ocio-economic status, **S**ocial capital and “Plus” that includes other context specific factors) provides a useful framework to contextualize the intersecting determinants of health in research design and program implementation [5, 6]. These items are often examined as confounders or effect modifiers in health research, but have received less attention in intervention studies, and are often not used to explicitly explore inequities in health.

Even though there is no exact definition of a disadvantaged or vulnerable group, human rights organisations have created comprehensive lists identifying characteristics of people who need special protection [7], many of which are PROGRESS Plus characteristics. As such, in this paper the terms disadvantage and vulnerability are used in relation to belonging to a group which may adversely affect your health and opportunities for health.

For the purposes of this paper, and within the larger scope of developing guidance for equity-relevant randomized trials [8], we will focus on the inequities that occur along the lines of the characteristics described in the PROGRESS Plus framework. We will focus on randomized controlled trials (RCTs), as they are often used in systematic reviews that inform guideline development and policy. In randomized controlled trials, the process of randomization creates (or is expected to create) prognostic balance between groups for measured and unmeasured confounders [9]. In some instances, reporting is disaggregated by PROGRESS Plus characteristics to demonstrate the differential effects they have on study outcomes. In other instances, PROGRESS Plus characteristics are used as subgroups in ancillary analyses to quantify the extent to which they affect outcomes. Often, only the best known sociodemographic variables such as, age, gender and level of education are considered—as these are known determinants of health [7], for which data are less sensitive and more readily available. As such these randomized trials may provide some, albeit limited information on items related to equity. Certain subgroups of patients experiencing disadvantages are excluded from trials because of their gender, age or ethnicity, and even when they are included, their sociodemographic details are not reported [10]. Pragmatic trials that include a broader scope of participants and address questions about the effectiveness of interventions in real world conditions [11], may be helpful in providing evidence on health inequities. It is plausible that with an a-priori focus on certain PROGRESS Plus characteristics, trials can be designed to optimise their ability to provide actionable and credible evidence on reducing inequities, by careful consideration of design, conduct and analytical issues that can help inform decisions about equity.

This piece of work finds its niche in the lack of guidance on how to design an equity-relevant trial. Few trials address (collect, analyse or report) all the PROGRESS Plus characteristics because not all of them are relevant to every outcome (or RCT) and many trials that may be equity-relevant do not purposefully seek to generate evidence on equity. In addition, PROGRESS Plus factors often interact with each other i.e. inequities can occur at multiple levels (for example low socioeconomic status may be linked to low level of education or differences in levels of education may be found to intersect across gender lines), and therefore careful thought should be given to how these nuances are captured. Given that trials are generally underpowered for subgroup analyses (which would elucidate the role of PROGRESS Plus characteristics) [12], it is important for investigators to recognise that subgroup data can be used in meta-analyses—potentially circumventing the lack of statistical power- if it is collected and reported adequately. In addition, certain interventions are known to aggravate inequalities in health [13]. The work presented in this paper is part of a collaborative effort to improve the design and reporting of equity-relevant trials, that includes the development of a Consolidated Standards of Reporting Trials (CONSORT) statement extension [8].

This paper is divided into three parts. The first provides guidance with respect to the formulation of research questions for equity-relevant trials and the second describes two different conceptual approaches to identifying equity relevant trials:

- A trial with a mixed population in which a PROGRESS Plus characteristic is a subgroup of interest.
- A trial that exclusively includes the disadvantaged group, defined based on one or more of the PROGRESS Plus characteristics.

The third part discusses some PROGRESS plus characteristics that may be considered in identifying potentially vulnerable populations included in RCTs.

## Formulating equity-relevant research questions

Many trials may report on some PROGRESS Plus characteristics (in the table describing the characteristics of the study participants or in the results) but were not necessarily designed with explicitly defined equity objectives in mind. The recommended approach to design an equity-relevant trial is to start with a well framed research question, where it is clear which equity-relevant characteristic(s) will be addressed. See the examples below which follow the PICO (Participants, Intervention, Comparison, Outcome) framework [14], with an equity item included (study design and timeframe not included for brevity).

- o Scenario A: Text messaging (I) versus usual care (C) in improving adherence to human immunodeficiency virus (HIV) medication (O) in people with HIV (P) of different age groups (equity item).
- o Scenario B: Text messaging (I) versus usual care (C) in improving adherence to HIV medication (O) in women with HIV (P defined across gender lines).

Current guidance would suggest that the discussion and conclusions from trials should focus on the overall treatment effect [12]. That reasoning may not apply to equity-relevant trials specifically aiming to explore the difference in effects between disadvantaged and non-disadvantaged groups in which the subgroup or disaggregated analyses is the purpose of the trial. In addition, secondary trial publications may have a clear focus on specific subgroups and thus provide equity-relevant evidence.

## Types of equity-relevant trials

### First scenario (A): A trial with a mixed population—equity (PROGRESS Plus) factor as a subgroup

Design considerations in scenario A will include powering the study for the subgroups of interest especially if these subgroups are included as part of the main study question. All relevant equity factors should be captured at baseline. Investigators might need to use additional techniques to ensure that randomization is balanced for important equity variables [9].

In brief, stratified randomization may be used to prevent imbalance between intervention groups for equity factors that may affect treatment outcomes. It will be most useful for small trials in which equity factors may have a large effect on outcomes. It ensures that equal numbers of participants are allocated to the intervention and control groups within each strata of the equity factor, and facilitates subgroup analyses [15]. For example, if we were concerned that gender would affect outcomes, we would use stratified randomization to ensure that among the males, equal numbers are randomized to intervention and control, and likewise among the females. In order to detect a subgroup effect as large as the overall treatment effect, the sample size should be inflated four-fold [16]. For smaller subgroup effects the inflation could be substantially larger and not always feasible. We still recommend that investigators collect information on these subgroups so that they can be used in adequately powered meta-analyses.

Analyses should be adjusted or stratified; with subgroup effects investigated using the appropriate approach. Drop-outs should be explored not only by intervention, but also by relevant equity factor. Further guidance has been published on these methodological issues [12, 17, 18].

### Second scenario (B): A trial that exclusively includes the disadvantaged group

Design considerations for Scenario B should include deep reflection about the purpose of the trial, such as whether it is ethical and appropriate to include (or exclude) this group of people experiencing a health inequity in a trial. Adequate justification must be given regarding the choice of population and strategies be implemented so that the trial does not aggravate pre-existing inequity. In fact, the trial should be adapted to accommodate the needs of the population experiencing inequities. Consulting with the community is recommended in such instances [19]. Given that the population of people to be included in the trial is sharing in a particular experience of health inequity (for example, they may have the same occupation or live in the same place), analysis should be straightforward, using techniques appropriate for RCTs. However, inequities may exist along multiple strata or PROGRESS Plus characteristics (for which data should be collected) and would need to be addressed if relevant. For example, an intervention may be less effective in males, with low levels of education and low income. In this instance, inequity exists along three strata—gender, education and income. These interactions can also be explored using the techniques for subgroup analyses described above (first scenario).

Noteworthy is the scenario in which the intervention is an attempt to address an equity issue. For example, financial incentives can affect socioeconomic status [20], educational interventions can reduce problems linked to low level of education [21], social capital interventions have been explored to improve mental health [22]. These sorts of interventions would naturally be conducted in people who are experiencing a disadvantage, and can be categorised as Scenario B.

**Fig. 1:**
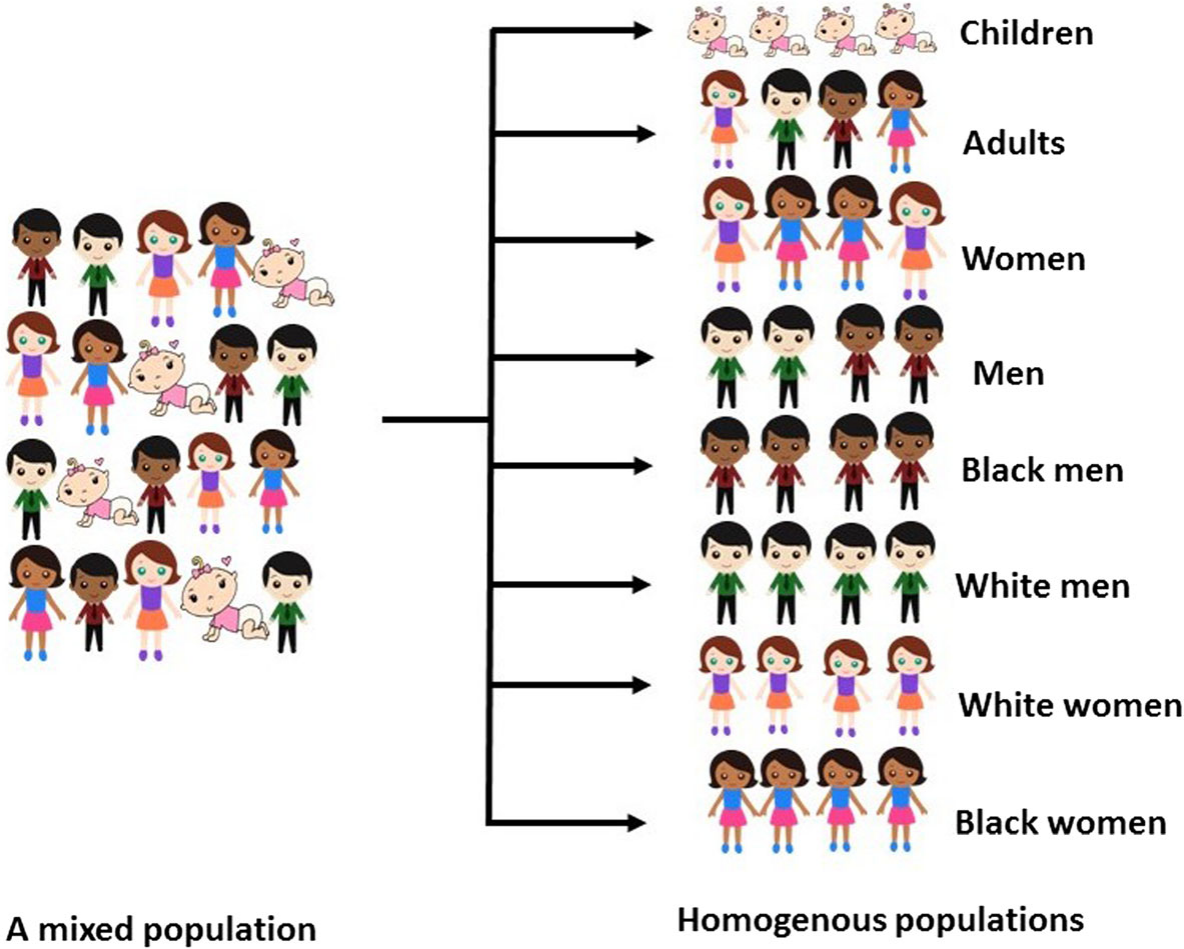
Mixed (scenario A) and homogenous population groups (scenario B—defined by age, gender and ethnicity). Courtesy of www.sweetclipart.com

Figure 1 is an illustration of a mixed population that can be split into homogenous groups.

### Rationale for considering various PROGRESS Plus characteristics for intervention studies

The premise of an equity-relevant trial design is that it aims to generate evidence about equity. For this to happen, the trial may include exclusively the groups experiencing inequity or be a mixture of both those experiencing and those not experiencing inequity such that comparative assessments can be made in disaggregated analyses or formal subgroup analyses. We believe all trials, especially those involving groups that experience some form of inequity should not aggravate these inequities. We emphasize the need to collect data on all relevant-equity factors. Some considerations in trial design, based on PROGRESS Plus characteristics are discussed below. We provide rationale for design choices that might affect external validity (due to exclusion of some groups of people), aggravate inequity (by causing undue hardships on some participants) or limit the use of trial data (by not collecting or reporting information relevant to inequities in health).

### Place of residence

Place of residence, as used here refers to any geographic differences in a trial participants’ habitat that could potentially influence inclusion in a trial, participation in a trial, or outcomes from a trial intervention. Typical categorisations of place of residence include rural versus urban, low versus high-income countries (differences may exist between and within countries) and others.

Trialists should consider how place of residence could affect an individual’s ability to attend study visits due to distance or other transportation issues, or if it will affect prognostic balance e.g. conducting a multicentre trial in areas of different malaria endemicity [23]. A well circumscribed place of residence can be a useful indicator of equity-relevance for the research project. However, typically high income places of residence may have pockets of poverty e.g. inner city districts.

#### Rationale

- o Inadequate consideration of place of residence may lead to exclusion of participants who live in remote or rural areas.
- o Outcomes might be different for participants who live in different geographical regions.
- o Losses to follow-up might be higher for participants who live far away from the trial site and may affect effect estimates.
- o Undue hardships may be imposed on participants having to travel too far for study visits.
- o Examples of trials addressing this issue: In this trial of a paraprofessional home-visiting intervention on American Indian teen mothers’ and infants’ behavioral risk, participants were recruited from four tribal reservation communities that were rural and isolated [24]. In a trial of mobile phone reminders to improve follow-up of medical care in children affected by HIV, the investigators deliberately targeted children in urban, semi-urban and rural areas [25].

### Race, ethnicity, culture and language

Trialists should consider the role of minority groups in the research question and ensure adequate representation of relevant groups. This can be done by involving communities in the design of the trial. These factors should be considered alongside other factors like place of residence, level of education and gender. Trialists should also acknowledge that a seemingly shared race, ethnicity and/or language do not necessarily generate homogenous groups of people. As well, the interplay of language and level of education may lead to reading ability being lower. Ethnicity may be relevant when considered with place of residence to reflect social capital (e.g. in immigrants).

#### Rationale

- o Excluding participants based on their ability to communicate in English potentially excludes minority groups, non-English speaking ethnicities and non-native English-speakers, and makes assumptions about the association between literacy and level of education. This has implications for generalizability. Trialists should not make assumptions about literacy and should consider translating consent forms and other trial reading material.
- o Ethnicity may also require further breakdown e.g. black could be African immigrant vs African American, two groups of people who might not be experiencing the same inequities.
- o Interventions that are meant to be applied to the general population should include adequate representation of these groups by ethnicity and language whenever possible. Translation of trial documents into multiple languages is appropriate for recruitment of diverse populations.
- o Examples of trials addressing this issue: A trial investigated a diabetes prevention intervention in Hispanics living in the Lower Yakima Valley, Washington, USA [26]. In another trial of protease inhibitor monotherapy compared to triple therapy for the reduction of viral load rebound in patients with HIV, ethnicity was found to affect virological rebound [27].

### Occupation

Occupation, viewed as a person’s role in society (student, employed, housewife etc.) or as a description of a person’s job (nurse, lawyer, teacher and plumber) is relevant in health research. It reflects, in most instances, level of education, access to resources, income, time available to participate in research and level of risk for certain diseases or chances of exposure to factors that affect health. In fact, certain diseases have been coined after some occupations, such as housemaids knee (prepatellar bursitis), miners’ lung (coal workers’ pneumoconiosis). Occupation may not always be relevant to the interventions or outcomes studied, but whenever it is, it should be adequately defined and categorised.

### Rationale

- o Baseline risk for certain conditions differs by occupation due to health damaging (e.g. exposure to asbestos) or health promoting exposures (e.g. wellness classes).
- o Occupation affects an individual’s ability to participate in research if they have less flexible work hours or less time available for participating in trials.
- o Examples of trials addressing this issue: A trial investigated a weight-loss intervention among truck drivers in the USA [28]. Another trial investigated interventions to promote the use of hearing protector devices in farm operators in the USA [29].

### Gender and Sex

Gender refers to the social construct while sex refers to the biological construct [30]. Considerable overlap exists in their use, as gender is the relationship of biological sex, gender identity and individuals' experience of gender roles. Gender or sex are typical groups for which data is collected in almost all heath research, but the analysis, reporting and interpretation of this data is often suboptimal [31].

#### Rationale

- o Many health outcomes differ based on fundamental biological differences between men and women; for example, Hemophilia is only expressed in men (women can be carriers). However, many differences in health outcomes exist due to the social differences between men and women [30].
- o Access to care, use of care and participation in research often differ by gender.
- o Gender may also be linked to other sources of inequity, like level of education and socio-economic status. Gender-based violence is known to disproportionately affect women [32].
- o Example: In one trial the investigators delivered a combined exercise and psycho-education intervention to reduce stress and depressive symptoms in Dutch women with low socio-economic status [33]. In another trial the metabolic changes to the traditional Mexican diet compared to a common US diet were investigated in women of Mexican descent in the USA [34].

### Religion (faith tradition)

Religion may affect participants’ ability to participate in an intervention or attend study visits on certain days. It may be a relevant prognostic factor for issues such as contraception use. It should be clearly defined (religion –system of belief with which an individual identifies versus religiosity- the practices that go along with that religion) [35], and may also be considered as a form of social capital (if regularly scheduled gatherings occur). It is also relevant to ethics board applications e.g. certain fertility and birth control interventions in Catholic institutions, blood transfusions in Jehovah’s witnesses’ groups. It may be appropriate to consult with local religious leaders on how to approach faith-based communities. Trialists must first be respective and engage local clergy and faith leaders to begin building relationships with the communities of interest, and be sensitive to historical harm that has affected minority communities of faith. The term “faith tradition” is increasingly being used in certain areas in the place of the term “religion” [36].

#### Rationale

- o Some religions encourage limited activity on 1 day of the week, and direct people in their daily practices
- o Religious gatherings may also be good places to share information and collect data from faith-based groups.
- o Religious fasting may affect study intervention and outcomes [37].
- o Religion and religiosity are known to affect health outcomes [38].
- o Example: In this trial a sexual health education intervention was assessed among married Muslim women in Iran [39].In another trial, the effect of motivational interviewing on alcohol and drug use was found to differ by religion among young adults in South Africa [40].

### Education

Education is usually a relevant factor that affects critical aspects of research studies such as enrollment, follow-up and adherence to study procedures or use investigational drugs. Sometimes, level of education is used as a screening criterion for inclusion in some trials. Investigators should consider adaptation of reading and listening material used to communicate with participants. Multimodal communication strategies, including reading, audio and video may be useful for populations with diverse educational backgrounds. Investigators should also consider the interplay of education and language. Plain language reading material may be challenging to use for people who do not speak the language. Level of education should be defined adequately (years of education vs levels completed; parallel educational paths like professional/technical education) to capture the issues investigated. Likewise, education may be related to socioeconomic status, so disentangling any independent effect of education may be difficult.

#### Rationale

- o Level of education affects participation in research, either due to restrictive inclusion criteria or inability to understand and provide consent.
- o It affects understanding of research procedures.
- o Example: In this trial of text messaging versus usual care to improve adherence to antiretroviral therapy, adherence was found to be better in people with a higher level of education [41]. In a trial of pharmaceutical care to improve treatment success in people living with HIV in Brazil, higher level of education was found to be predictive of virological success [42].

### Socio-economic status

Socioeconomic status is typically relevant. Unfortunately, it is often challenging to capture in a comprehensive way. It may be influenced by age, gender, place of residence, education and ethnicity. Composite scores (include housing, transport, cooking and toilet facilities, water sources etc.) should be preferred over income alone [43].

#### Rationale

- o When measured in a more inclusive manner, accounting for all relevant factors that determine socioeconomic status (composite scores), it may be a very good reflection of baseline risk for disease.
- o It reflects access to resources, including health care.
- o Example: In this trial, the investigators tested physical exercise and psycho-education for the reduction of stress among women of low socio-economic status in the Netherlands [33]. Another trial investigated a school-based nutritional intervention in low-socioeconomic school children in Israel [44].

### Social capital

Social capital is an important factor in psychosocial research which refers to social relationships and networks [5],however it is hard to define and measure as it encompasses many dimensions [45]. It is positively associated with a range of beneficial social, economic and health outcomes [46]. It interacts with other PROGRESS Plus characteristics: age, gender, religion, ethnicity/place of residence (consider displaced persons i.e. migrants and refugees).

#### Rationale

- o Psycho-social support is an important part of health care and patient wellbeing.
- o It is known to affect certain health outcomes.
- o It may be a health outcome.
- o Example: In this trial the investigators explored the effects of cognitive therapy on social capital in survivors of sexual violence in the Democratic Republic of Congo [47]. In another trial, the investigators explored strategies to improve social capital in Limpopo, South Africa [46].

## Discussion

In the preceding pages, we have noted some key considerations for investigators conducting equity-relevant trials, notably in how to frame the research question so that the equity-focus is clear, two useful approaches to including participants in equity-relevant trials and the role that each of the PROGRESS Plus factors can play in the design and conduct of equity-relevant trials.

We acknowledge that not all PROGRESS Plus factors will be relevant to all trials and that collecting data on these factors may lead to longer questionnaires, more time to collect data, a higher burden on the participant and more costly trials. More so, not all PROGRESS Plus characteristics directly imply that the groups are disadvantaged. We invite investigators to consider the potential relevance of each of these characteristics to their research and how this information on PROGRESS Plus characteristics that is collected is going to be used. Community-based participatory research approaches have the potential to address some of the issues raised in this paper [48], especially with regards to vulnerable populations. Understanding how to conduct research with so called “hard-to-reach” populations is a recognised barrier to building important research evidence that can address socially perpetuated inequities, and various sampling techniques have been developed to include participants who are culturally, socially, economically or geographically so called “hard-to-reach” [49].

There is evidence that many groups of people are excluded from trials. For example, the elderly, females and minorities are often excluded from heart failure trials [50], older people are underrepresented in drug trials [51] and people living with HIV are often unjustifiably excluded from lymphoma trials [52]. Whether these exclusions are well-founded or not, they often limit the generalisability of the trial results, and would be considered unfair especially if the populations excluded shoulder a disproportionate burden of disease.

Equally relevant is due consideration on how the equity factors will be addressed. The first scenario (Scenario A) in which heterogeneous populations are included requires a larger sample, but is a good opportunity to explore contrast in outcomes across multiple equity factors. Findings from such a trial are more likely to be generalizable and will provide information relevant to both disadvantaged and non-disadvantaged groups. On the other hand, for well-established inequities, it may no longer be useful to explore the contrast, but rather the effect of the intervention in the disadvantaged group (Scenario B). In this instance, the disadvantaged group can be defined across one or more equity factors. The more characteristics used to define the study population, the more unique it becomes. Such a trial is likely to provide a robust source of information on the group included in the study. Some examples include: an intervention to improve posture and weight among women (gender characteristic) above 50 years of age (age characteristic) with sedentary occupations (occupation factor) [53], and a trial investigating paraprofessional home-visiting on American Indian (ethnicity characteristic) teen (age characteristic) mothers (gender characteristic) living on rural isolated tribal communities (place of residence characteristic) on infants’ behavioral risks [24].

Some trials may report evidence on equity, even though the trialists did not purposefully set out to do so. For example, in the Cameroon Mobile Phone SMS (CAMPS) trial using mobile phone text messages to improve adherence to antiretroviral therapy, the investigators found that the intervention was more likely to work in people with higher levels of education [41]. We believe that incorporating equity-design considerations at the planning stages will optimise the collection, analyses and reporting of equity-relevant data that can inform future trial design, policy and implementation.

## Conclusion

By carefully framing the research question, selecting the most appropriate population group and assessing the role of equity factors in the design and analyses of equity-relevant trials, trialists can contribute to developing a robust body of evidence on the effects of interventions in disadvantaged groups.

## Abbreviations

CAMPS: Cameroon Mobile Phone SMS trial
CONSORT: Consolidated Standards of Reporting Trials
HIV: Human Immunodeficiency Virus
PICO: Acronym for Participants, Intervention, Comparator, Outcome
PROGRESS: Acronym for Place of residence, Race/ethnicity, Occupation, Gender, Religion, Education, Socioeconomic status, Social capital
RCT: Randomized Controlled Trial
USA: United States of America
